# Management of *Helicobacter pylori* infection in paediatric patients in Europe: results from the EuroPedHp Registry

**DOI:** 10.1007/s15010-022-01948-y

**Published:** 2022-11-03

**Authors:** Thu Giang Le Thi, Katharina Werkstetter, Kallirroi Kotilea, Patrick Bontems, José Cabral, Maria Luz Cilleruelo Pascual, Michal Kori, Josefa Barrio, Matjaž Homan, Nicolas Kalach, Rosa Lima, Marta Tavares, Pedro Urruzuno, Zrinjka Misak, Vaidotas Urbonas, Sibylle Koletzko, for the Helicobacter pylori Special Interest Group of ESPGHAN, Josef Sykora, Erasmo Miele, Andreas Krahl, Martina Klemenak, Alexandra Papadopoulou, Andrea Chiaro, Meltem Korkut Ugras, Jan de Laffolie, Krzysztof Matusiewics, Francesca Rea, Thomas Casswall, Eleftheria Roma, Hany Banoub, Aron Cseh, Maria Rogalidou, Ana Isabel Lopes

**Affiliations:** 1 Dr. von Hauner Children’s Hospital, University Hospital, LMU Munich, Lindwurmstrasse 4, 80337 Munich, Germany; 2 Université Libre de Bruxelles, Hôpital Universitaire des Enfants Reine Fabiola, Brussels, Belgium; 3 Pediatric Gastroenterology Unit, Dona Estefânia Hospital, University Hospital Centre of Central Lisbon, Lisbon, Portugal; 4 Pediatrics Department, Gastroenterology Unit, University Hospital Puerta de Hierro Majadahonda, Madrid, Spain; 5 Pediatric Gastroenterology, Kaplan Medical Centre, Rehovot, Israel; 6 Faculty of Medicine, Hebrew University of Jerusalem, Jerusalem, Israel; 7 Department of Paediatrics, Hospital Universitario de Fuenlabrada, Madrid, Spain; 8 Department of Gastroenterology, Hepatology, and Nutrition, Faculty of Medicine, University Children’s Hospital, University of Ljubljana, Ljubljana, Slovenia; 9 Groupement des Hôpitaux de l’Institut Catholique de Lille (GHICL), Saint Antoine Paediatric Clinic, Saint Vincent de Paul Hospital, Catholic University, Lille, France; 10 Division of Paediatrics, Paediatric Gastroenterology Department, Centro Materno Infantil do Norte, Centro Hospitalar Universitário do Porto, ICBAS - Instituto de Ciências Biomédicas Abel Salazar, Porto, Portugal; 11 Pediatric Gastroenterology Unit, Hospital 12 de Octubre, Madrid, Spain; 12 Referral Centre for Paediatric Gastroenterology and Nutrition, Children’s Hospital Zagreb, University of Zagreb School of Medicine, Zagreb, Croatia; 13 Clinic of Children’s Diseases of Vilnius University Faculty of Medicine, Vilnius, Lithuania; 14 Department of Paediatrics, Gastroenterology and Nutrition, School of Medicine Collegium Medicum University of Warmia and Mazury, Olsztyn, Poland

**Keywords:** *Helicobacter pylori*, *Helicobacter pylori*—in children, Antibiotic therapy, Drug resistance, Paediatric gastroenterology

## Abstract

**Purpose:**

The EuroPedHp-registry aims to monitor guideline-conform management, antibiotic resistance, and eradication success of 2-week triple therapy tailored to antibiotic susceptibility (TTT) in *Helicobacter pylori-*infected children.

**Methods:**

From 2017 to 2020, 30 centres from 17 European countries reported anonymized demographic, clinical, antibiotic susceptibility, treatment, and follow-up data. Multivariable logistic regression identified factors associated with treatment failure.

**Results:**

Of 1605 patients, 873 had follow-up data (53.2% female, median age 13.0 years, 7.5% with ulcer), thereof 741 (85%) treatment naïve (group A) and 132 (15%) after failed therapy (group B). Resistance to metronidazole was present in 21% (*A*: 17.7%, *B*: 40.2%), clarithromycin in 28.8% (*A*: 25%, *B*: 51.4%), and both in 7.1% (*A*: 3.8%, *B*: 26.5%). The majority received 2-week tailored triple therapy combining proton pump inhibitor (PPI), amoxicillin with clarithromycin (PAC) or metronidazole (PAM). Dosing was lower than recommended for PPI (*A*: 49%, *B*: 41%) and amoxicillin (*A*: 6%, *B*: 56%). In treatment naïve patients, eradication reached 90% (*n* = 503, 95% CI 87–93%) and 93% in compliant children (*n* = 447, 95% CI 90–95%). Tailored triple therapy cured 59% patients after failed therapy (*n* = 69, 95% CI 48–71%). Treatment failure was associated with PAM in single clarithromycin resistance (OR = 2.47, 95% CI 1.10–5.53), with PAC in single metronidazole resistance (OR = 3.44, 95% CI 1.47–8.08), and with low compliance (OR = 5.89, 95% CI 2.49–13.95).

**Conclusions:**

Guideline-conform 2-weeks therapy with PPI, amoxicillin, clarithromycin or metronidazole tailored to antibiotic susceptibility achieves primary eradication of ≥ 90%. Higher failure rates in single-resistant strains despite tailored treatment indicate missed resistance by sampling error.

**Supplementary Information:**

The online version contains supplementary material available at 10.1007/s15010-022-01948-y.

## Background

*Helicobacter pylori (H. pylori)* infections are mostly acquired in early childhood [1]. *H. pylori* infection causes chronic gastritis, although most children remain asymptomatic [2, 3]. Eradication of *H. pylori* infection improves gastric inflammation and reduces the risk for recurrent peptic ulcer disease (PUD) and malignancies [4]. *H. pylori* treatment should aim to reach a high primary eradication rate of at least 90% [5–8].

In the past years, the unnecessary and inappropriate use of antibiotics has led to high antibiotic resistance, including those used for *H. pylori* treatment (e.g. clarithromycin, metronidazole, and levofloxacin) [9–11]. In 2017, the World Health Organization designated clarithromycin-resistant *H. pylori* as a high-priority bacterium for antibiotic research and development [10]. In the era of increasing antibiotic resistance and decreasing eradication success, *H. pylori* therapies should be based on antimicrobial stewardship principles optimizing antibiotic use while reducing antibiotic resistance [5, 6]. Graham and Liou 2021 emphasized the importance of treatment tailored to antibiotic susceptibility regardless of age. Only antibiotics susceptible to infecting strains should be prescribed [5, 6], acknowledging that at least two-thirds of the *H. pylori* strains become resistant after treatment failure [12, 13].

In 2016, the European and North American Societies of Paediatric Gastroenterology, Hepatology and Nutrition (ESPGHAN, NASPGHAN) updated guidelines to reach an eradication success of at least 90% in treatment naïve patients. They recommend first-line triple therapy with body weight-adjusted dosing combining proton-pump-inhibitor (PPI) at a higher dose (maximum 80 mg/day (es)-omeprazole or equivalent) plus two antibiotics tailored to susceptibility testing for 2 weeks [7, 8]. Sequential therapy is restricted to fully susceptible strains [3, 8, 14]. A higher daily amoxicillin (AMO) dose (maximum 3000 mg instead of 2000 mg) is recommended for infections with double-resistant strains or after treatment failure [8]. In children, a high initial eradication rate is crucial to avoid repetitive courses of antibiotics with the risk of inducing dysbiosis and antibiotic resistance. A high initial success rate will decrease repeated investigations (e.g. endoscopies), therapies and, consequently, costs and burdens for the patient, their families, and society [7].

Data on antibiotic resistance and treatment outcomes in children and adolescents living in Europe are sparse, and most are restricted to single centres [15, 16]. The *H. pylori* working group of the ESPGHAN initiated the EuroPedHp registry to survey antibiotic resistance, compliance to guideline-conform treatments, and eradication rate (ER) of the recommended treatment regimen. The data gathered from 2017 to 2020 allow us to investigate factors associated with treatment failure of tailored triple therapy (TTT). Furthermore, in countries with available bismuth-based therapy (BMT), we survey the cure rate of BMT in patients with double resistance to clarithromycin and metronidazole or after failed therapy.

## Methods

### Design and data collection

The EuroPedHp registry started in 2013 with the main aim of surveillance of antibiotic resistance [17]. From January 2017 onwards, data collection was extended for clinical and endoscopic findings, prescribed treatment, compliance, and therapy success. Participating centres from 17 European countries, including Israel and Turkey, anonymously submitted information on *H. pylori*-infected paediatric patients on demographics (age, gender, country of birth from patient and their parents), symptoms and other indications leading to upper-endoscopy, previous anti-*H. pylori* therapies, co-morbidities, endoscopic findings, antibiotic susceptibility testing, prescribed therapy and compliance with drug intake, adverse events during and after treatment and assessment of treatment success. Regarding country of living or country of birth, we assigned countries to four European geographical regions (Northern, Western, Southern, and Eastern) and outside Europe, including Israel, Turkey, the Middle East, Asia, Africa, and America (Supplementary File 1). *H. pylori* infection was confirmed according to guidelines [7, 8] by a positive culture or positive results of two other tests, either biopsy-based (histopathology, rapid urease test, or RT-PCR) or noninvasive [^13^C-urea breath test (UBT) or monoclonal stool antigen test (SAT)].

Antibiotic susceptibility of *H. pylori* strains for clarithromycin, metronidazole, amoxicillin and second-line antibiotics like tetracycline, levofloxacin, and rifampicin was assessed at the local centres by using epsilometer test (E-test) or disc diffusion, occasionally real-time polymerase chain reaction (RT-PCR).

The local paediatric gastroenterologist decided on the treatment regimen, type, dose (mg/kg) and duration of applied PPI and antibiotics. Participating centres were encouraged to follow the evidence-based guidelines for the management of *H. pylori* infection in children and adolescents published in 2011 [7] and updated in 2017 (online available in 2016) [8] by the ESPGHAN/NASPGHAN and to prescribe the recommended dosing regimens according to body weight (Supplementary File 2).

Eradication success was assessed by noninvasive tests (UBT or monoclonal SAT) or gastric biopsies at least 4 weeks after completed treatment. Eradication rate (ER) was evaluated as the proportion of all patients treated successfully with a confirmed negative test relative to all treated patients with follow-up results.

Strict monitoring of treatment success was highly recommended. In cases of failed treatment, therapeutic regimens were chosen based on antibiotic susceptibility results, patient age, and availability of bismuth-containing drugs or other reserve antibiotics.

### Data reporting and centre monitoring

Patient records were anonymously submitted in an electronic case report form (e-CRF) (Castor EDC, Amsterdam, The Netherlands). At the annual meetings of *H. pylori* working group of the ESPGHAN, interim data of the EuroPedHp registry were critically reviewed. Participating centres received newsletters providing practical recommendations to improve treatment success.

The protocol for irreversibly anonymized data collection was approved by the Ethical Committee of the LMU University Hospital Munich, Germany (project number: 105–13). Participating centres achieved approval from their local ethical committee. The registry was financially supported by the ESPGHAN and by research funds of Prof. Dr med. Sibylle Koletzko, LMU-Klinikum Munich, Germany.

### Statistical analysis

Descriptive statistics for demographical and clinical characteristics are presented in two groups: patients prior to first anti-*H. pylori* therapy (treatment naïve) (group A) and after at least one failed therapy (group B).

The final analysis set (FAS) contains all paediatric patients with proven *H. pylori* infection, who received anti-*H. pylori* therapy, took at least one treatment dose and completed follow-up. Per-protocol (PP) analysis included all patients in the final analysis set who took at least 90% of prescribed drugs. Drug doses calculated by three weight classes (Supplementary File 2) were classified as “conform with guidelines” or “lower than recommended” [8].

To determine statistically significant differences between groups, we performed Mann–Whitney *U*-test for continuous variables, while Pearson’s Chi-square test or Fisher’s exact test for categorical variables where appropriate. All statistical tests were assessed with two-sided significance levels of 5%.

A univariate logistic analysis was performed to determine potential risk factors for treatment failure. Using the same samples as in the univariate analysis, the final multivariable logistic models were selected using backward elimination and adjusted for gender and age (in years) (Supplementary File 3). Estimated odds ratio (OR) and 95% confidence interval (CI) were reported.

To determine the difference between the real-life data and data including only cases treated with guideline-conform choices of antibiotics, we performed a sensitivity analysis after excluding cases where physicians falsely prescribed antibiotics to patients whose strains were resistant to.

Statistical analyses were performed using the SAS program (Statistical Analysis Software 9.4, SAS Institute Inc., Cary, North Carolina, USA) and Prism 9.3 (GraphPad Software).

## Results

### Study population

From 2017 to 2020, 1543 valid records of 1605 patients were reported by 30 centres from 17 European countries, thereof all but 15 children had undergone upper endoscopies with biopsies in the reporting centres. Treatment against *H. pylori* infection was prescribed to 1263 patients, and thereof 873 completed follow-up (Supplementary File 4). Reasons for untreated cases include detection of *H. pylori* infection by chance at endoscopy for other disorders, no symptoms, young age, or parents’ refusal. One-third of treated patients did not return for monitoring the success of therapy.

The baseline characteristics of the final analysed cohort are presented in Table 1 (53.2% female, median age: 13.0 years) with 741 of 873 (85%) treatment naïve patients (group A) and 132 (15%) patients after failed therapy (group B). A high proportion of reported patients live in Southern Europe, predominantly in Spain and Portugal (Table 1, Supplementary File 5).

**Table 1:** Basic characteristics of *H. pylori*-infected paediatric patients in the EuroPedHp registry from 2017 to 2020 for the total cohort with follow-up data (final analysis set, FAS, *N* = 873) (*IQR* interquartile range, *MET* metronidazole, *CLA* clarithromycin)

Factors, *n* (%)	All patients *N* = 873 (100%)	Group A treatment naïve patients, *n* = 741 (85%)	Group B patients after failed therapy, *n* = 132 (15%)	*p* value^a^
Demographics				
Gender—female	464 (53.2)	390 (52.6)	74 (56.1)	0.467
Age (years), median (IQR)	13.0 (10.3–15.2)	13.0 (10.3–15.1)	12.8 (10.2–15.4)	0.352
Age group (years), *n* = 873				0.573
Age < 12	351 (40.2)	295 (39.8)	56 (42.4)	
Age ≥ 12	522 (59.8)	446 (60.2)	76 (57.6)	
Weight, *n* = 863, median (IQR)	45.5 (34.0–57.5)	45.8 (34.0–57.5)	45.0 (33.0–57.3)	0.712
Weight groups (kg), *n* = 863				0.916
< 25	85 (9.8)	72 (9.8)	13 (9.9)	
25–34	141 (16.3)	118 (16.1)	23 (17.6)	
> 35	637 (73.8)	542 (74.0)	95 (72.5)	
Country of living^b^, *n* = 873				** < 0.0001**
Northern/Western Europe	232 (26.6)	204 (27.5)	28 (21.2)	
Southern Europe	419 (48.0)	364 (49.1)	55 (41.7)	
Eastern Europe	164 (18.8)	140 (18.9)	24 (18.2)	
Israel and Turkey	58 (6.6)	33 (4.5)	25 (18.9)	
Country of birth^b^, *n* = 777				**0.002**
Northern/Western Europe	173 (22.3)	153 (23.4)	20 (16.4)	
Southern Europe	317 (40.8)	273 (41.7)	44 (36.1)	
Eastern Europe	175 (22.5)	148 (22.6)	27 (22.1)	
Asia, Africa, America and Middle East	112 (14.4)	81 (12.4)	31 (25.4)	
Mother’s country of birth^b^, *n* = 719				0.371
Northern/Western Europe	35 (4.9)	28 (4.6)	7 (6.0)	
Southern Europe	260 (36.2)	223 (37.0)	37 (31.9)	
Eastern Europe	189 (26.3)	162 (26.9)	27 (23.3)	
Asia, Africa, America and Middle East	235 (32.7)	190 (31.5)	45 (38.8)	
Symptoms associated with *H. pylori* infection				
Abdominal pain	667 (76.6)	552 (74.7)	115 (87.1)	**0.002**
Nausea	137 (15.7)	112 (15.2)	25 (18.9)	0.271
Vomiting	134 (15.4)	111 (15.0)	23 (17.4)	0.481
Bloating	46 (5.3)	39 (5.3)	7 (5.3)	0.99
Diarrhoea	34 (3.9)	30 (4.1)	4 (3.0)	0.574
Constipation	29 (3.3)	28 (3.8)	1 (0.8)	0.108
Metallic taste	4 (0.5)	3 (0.4)	1 (0.8)	0.482
Endoscopic findings				
Endoscopy at presenting centre, *n* = 873	865 (99.1)	733 (98.9)	132 (100)	0.230
Year of endoscopy, *n* = 865				0.506
2017	336 (38.8)	279 (38.1)	57 (43.2)	
2018	300 (34.7)	256 (34.9)	44 (33.3)	
2019 and 2020	229 (26.5)	198 (27.0)	31 (23.5)	
Primary indication for endoscopy, *n* = 865				** < 0.0001**
Abdominal pain	578 (66.8)	476 (64.9)	102 (77.3)	
Dyspepsia incl. nausea, vomiting	93 (10.8)	76 (10.3)	17 (13)	
Anaemia	36 (4.2)	35 (4.8)	1 (0.8)	
GastrointestinaI-bleeding	18 (2.1)	18 (2.5)	0	
Celiac disease	27 (3.1)	27 (3.7)	0	
Eosinophilic esophagitis	24 (2.8)	21 (2.9)	3 (2.3)	
Inflammatory bowel disease	10 (1.2)	10 (1.4)	0	
Others: weight loss, diarrhoea, etc	68 (7.9)	65 (8.9)	3 (2.3)	
Only positivity in noninvasive tests	11 (1.3)	5 (0.7)	6 (4.5)	
Number of biopsies, *n* = 843, median (IQR)	4 (4–6)	4 (4–6)	5 (4–6)	**0.012**
Antral nodularity, *n* = 863	737 (85.4)	622 (84.9)	115 (88.5)	0.283
Suspected eosinophilic esophagitis, *n* = 862	47 (5.4)	36 (4.9)	11 (8.4)	0.107
Ulcers, *n* = 862	65 (7.5)	57 (7.8)	8 (6.2)	0.532
Erosions, *n* = 862	141 (16.4)	117 (16.0)	24 (18.6)	0.454
Positive rapid urease test (RUT), *n* = 342	310 (90.6)	270 (90.3)	40 (93.0)	0.567
Histology confirmed, *n* = 500^c^	465 (93.0)	410 (94.3)	55 (84.6)	**0.017**
Susceptibility testing, *n* = 873				**0.0491**
Culture positive and/or PCR available	775 (88.8)	666 (89.9)	109 (82.6)	
Culture negative and no PCR	37 (4.2)	28 (3.8)	9 (6.8)	
Not applicable or unknown	61 (7.0)	47 (6.3)	14 (10.6)	
Antibiotic resistance profile				
Metronidazole resistance, *n* = 706	148 (21.0)	107 (17.7)	41 (40.2)	** < 0.0001**
Clarithromycin resistance^d^, *n* = 760	219 (28.8)	163 (25.0)	56 (51.4)	** < 0.0001**
Amoxicillin resistance, *n* = 662	10 (1.5)	6 (1.0)	4 (4.9)	**0.007**
Tetracycline resistance, *n* = 584	3 (0.5)	2 (0.4)	1 (1.5)	0.227
Levofloxacin resistance^d^, *n* = 664	35 (5.3)	31 (5.4)	4 (4.3)	0.652
Rifampicin resistance, *n* = 172	14 (8.1)	12 (8.5)	2 (6.7)	0.745
Metronidazole and clarithromycin resistance—Susceptibility subgroups^e^, *n* = 706				** < 0.0001**
MET-Susceptible/CLA-Susceptible	406 (57.5)	370 (61.3)	36 (35.3)	
MET-Susceptible/CLA-Resistant	152 (21.5)	127 (21.0)	25 (24.5)	
MET-Resistant/CLA-Susceptible	98 (13.9)	84 (13.9)	14 (13.7)	
MET-Resistant/CLA-Resistant	50 (7.1)	23 (3.8)	27 (26.5)	

Results were presented in median and interquartile range (IQR) from 25% quartile to 75% quartile for continuous variables and in frequency (*n*) and column percentage (%) for categorical variables

^a^*P* values obtained by Mann–Whitney *U*-test for continuous variables, while Pearson’s Chi-square test or Fisher’s exact test for categorical variables as appropriate. Bold *p* values indicate significant differences in the proportion of respective factors between group A (treatment naïve patients) and group B (patients after failed therapy) with a *p* value ≤ 0.05

^b^Country distribution was given in supplementary file 2

^c^Data of histology were collected from 2018 to 2020

^d^Data were collected from thereof real-time polymerase chain reaction (RT-PCR) test

^e^Data are based on all available susceptibility test results for metronidazole (MET) and clarithromycin (CLA)

### Clinical presentation

Endoscopy was performed in almost all cases (99.1%, *n* = 873). Abdominal pain was the primary indication for endoscopy in 66.8% (*n* = 865, Table 1). Macroscopic findings disclosed antral nodularity in 85.4% of infected children, while 7.5% showed gastric and/or duodenal peptic ulcers and 16.4% erosions. Macroscopic signs of eosinophilic esophagitis were observed in 5.4% (Table 1).

### Antibiotic susceptibility results

Antibiotic susceptibility results were available in 710 of 727 with positive culture and in 131 patients from RT-PCR testing. Primary resistance (group A) to metronidazole or clarithromycin was found in 17.7% (*n* = 604, 95% CI 14.7–20.8%) and 25.0% (*n* = 651, 95% CI 21.7–28.4%) of strains, respectively (Table 1). Once patients had failed *H. pylori* therapy (group B), the resistance rate increased to 40.2% (*n* = 102, 95% CI 30.7–49.7%) against metronidazole and to 51.4% (*n* = 109, 95% CI 42.0–60.8%) against clarithromycin. Strains susceptible to both clarithromycin and metronidazole were present in 57.5% (*A*: 61.3%, *B*: 35.3%), while double resistance to both clarithromycin and metronidazole was reported in treatment naïve patients 3.8% (*n* = 604, 95% CI 2.3–5.3%), but increased in patients after failed therapy to 26.5% (*n* = 102, 95% CI 17.9–35.0%). Resistance to amoxicillin was rare (*n* = 662, *A*: 1.0%, *B*: 4.9%).

The primary resistance rate to levofloxacin and rifampicin was found in 5.4% (*n* = 571) and 8.5% (*n* = 142), respectively, with few cases having documented resistance to tetracycline (Table 1).

### Eradication success of common treatment regimens

**Fig. 1:**
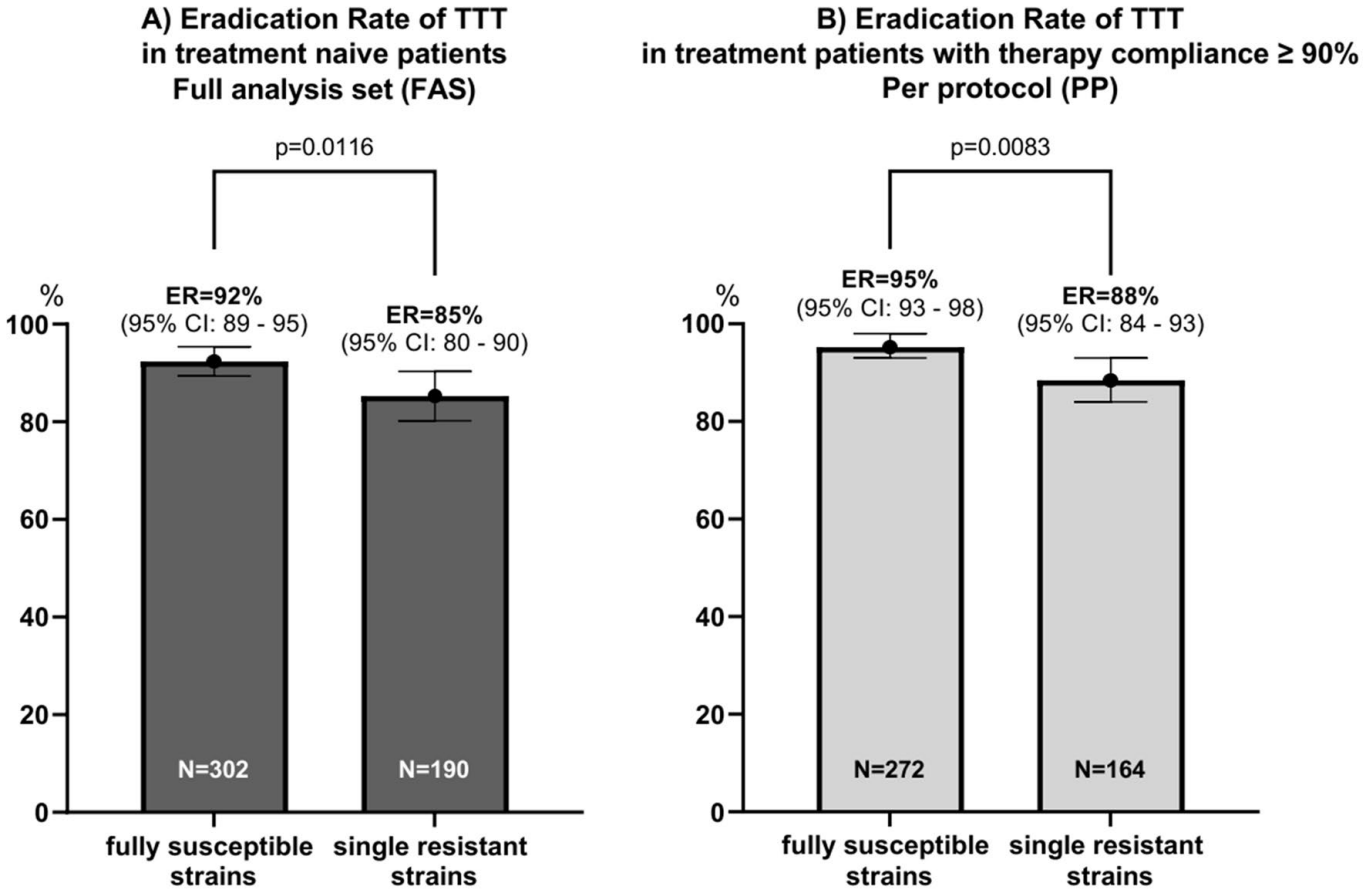
**A** and **B** Eradication rate (ER) of tailored triple therapy (TTT) in patients with fully susceptible strains vs single-resistant strains. **A** In full analysis set (FAS). **B** Per protocol (PP) population. Abbreviation: *TTT* tailored triple therapy, *ER* eradication rate, *FAS* full analysis set, *PP* per protocol. ER% represents eradication rate (ER) in per cent (%) as the proportion of all patients treated successfully with a confirmed negative test after completed treatment relative to all patients treated in a specific sub-group. *P* values were obtained from Pearson’s Chi-square test to determine the significant difference in eradication rate (ER) between patient group infected with fully susceptible strains to both clarithromycin and metronidazole vs patient group infected with single-resistant strains to clarithromycin or metronidazole

Two weeks of tailored triple therapy with PPI, amoxicillin, clarithromycin (PAC) or PPI, amoxicillin, metronidazole (PAM) were prescribed to 80.4% (702/873), 10% each received sequential (*n* = 86) or other therapy regimens (*n* = 85). Tailored triple therapy cured the infection in 90% of treatment naïve children (*n* = 503, 95% CI 87–93%) and in 93% in naïve patients adhering to therapy (per-protocol analysis) (*n* = 447, 95% CI 90–95%) (Table 2). Eradication rate was higher in compliant patients than in less compliant patients (ER = 93% vs ER = 63%, *p* < 0.0001). Infected children with fully susceptible strains achieved a significantly higher eradication rate than those harbouring single-resistant strains, both in the final analysis set and the per-protocol population (Fig. 1).

**Table 2:** Eradication rate (ER) of the most common treatment regimens in relation to antibiotic susceptibility in treatment naïve patients (group A) and patients after failed therapy (group B)

Susceptibility sub-groups/common treatments	All subgroups ER% (*n*/*N*)	MET-S/CLA-S ER% (*n*/*N*)	MET-S/CLA-R ER% (*n*/*N*)	MET-R/CLA-S ER% (*n*/*N*)	MET-R/CLA-R ER% (*n*/*N*)
Group A (treatment naïve patients)					
Tailored triple therapy (TTT) including PAC and PAM	90% (452/503)	92% (279/302)	86% (94/109)	84% (68/81)	100% (11/11)
PPI + AMO + CLA (PAC)	88% (252/285)	91% (185/203)	0% (0/2)	83% (65/78)	100% (2/2)
PPI + AMO + MET (PAM)	92% (200/218)	95% (94/99)	88% (94/107)	100% (3/3)	100% (9/9)
PPI + AMO + CLA + MET sequential	82% (50/61)	86% (44/51)	56% (5/9)	100% (1/1)	N.A
Group B (patients after failed therapy)					
Tailored triple therapy (TTT) including PAC and PAM	59% (41/69)	65% (22/34)	71% (15/21)	30% (3/10)	25% (1/4)
PPI + AMO + CLA (PAC)	52% (14/27)	60% (12/20)	N.A	29% (2/7)	N.A
PPI + AMO + MET (PAM)	64% (27/42)	71% (10/14)	71% (15/21)	33% (1/3)	25% (1/4)
PPI + AMO + other antibiotic(s)	69% (9/13)	N.A	100% (2/2)	100% (1/1)	60% (6/10)
Bismuth-based therapy (BMT)	83% (10/12)	N.A	N.A	100% (2/2)	80% (8/10)

Data are based on all available susceptibility test results for metronidazole (MET) and clarithromycin (CLA)

ER% represents eradication rate (ER) in per cent (%) as the proportion of all patients treated successfully with a confirmed negative test after completed treatment (*n*) relative to all patients treated (*N*)

Abbreviation: *TTT* tailored triple therapy, *ER* eradication rate, *PPI* proton pump inhibitor, *AMO* amoxicillin, *CLA* clarithromycin, *MET* metronidazole, *PAC* for treatment regimen with proton pump inhibitor, amoxicillin, clarithromycin, *PAM* for treatment regimen with proton pump inhibitor, amoxicillin, metronidazole, *BMT* bismuth-based therapy, *N.A.* not applicable

MET-S/CLA-S: Strains susceptible to both metronidazole and clarithromycin. MET-S/CLA-R: Strains susceptible to metronidazole but resistant to clarithromycin. MET-R/CLA-S: Strains resistant to metronidazole but susceptible to clarithromycin. MET-R/CLA-R: Strains resistant to both metronidazole and clarithromycin

PAM showed a trend for higher success than PAC, in both fully susceptible (ER = 95% vs ER = 91%, *p* = 0.241) and single-resistant strains (ER = 88% vs ER = 83%, *p* = 0.383) (Table 2, Supplementary 6). Sequential therapy (SQT), although mostly used in fully susceptible patients, did not reach the 90% goal (ER = 86%, *n* = 51, 95% CI 77–96%).

Tailored triple therapy in patients with previously failed therapy (group B) performed poorly (ER = 59%, *n* = 69, 95% CI 48–71%), while bismuth-based therapy was successful in 80% (*n* = 15), including in eight of ten children harbouring double-resistant strains (Table 2).

The most commonly prescribed PPIs were omeprazole or esomeprazole (92%) and the remaining lansoprazole (2%) or pantoprazole (6%). The PPI dose was lower than recommended in the guidelines [8] in half of all patients (Table 3, Supplementary File 7). Antibiotic doses were prescribed according to guidelines in 80% of the patients, except in patients after failed therapy, in which more than half received lower amoxicillin doses than recommended (Supplementary File 7).

**Table 3:** Risk factors for eradication failure of tailored triple therapy (TTT) among treatment naïve patients (group A), *N* = 503

Factors, *n* (row percent %)	*N*	ER failed *n* (%)	ER success *n* (%)	*p* value^a^	OR_crude_^b^ (95% CI)	*p* value^c^	OR_adj_^d^ (95% CI)	*p* value^c^
Gender				0.520				
Female	268	25 (9%)	243 (91%)		Ref			
Male	235	26 (11%)	209 (89%)		1.21 (0.68–2.16)	0.520		
Age (years), median (IQR)	503	13 (11–15)	13 (10–15)	0.871	1.03 (0.94–1.13)	0.546		
Country of living^e^				0.288				
Northern/Western Europe	146	11 (8%)	135 (92%)		Ref			
Southern Europe	222	21 (9%)	201 (91%)		1.28 (0.60–2.75)	0.522		
Eastern Europe	113	16 (14%)	97 (86%)		2.02 (0.90–4.55)	0.088		
Israel and Turkey	22	3 (14%)	19 (86%)		1.94 (0.50–7.58)	0.342		
Susceptibility sub-groups^f^				0.053				
MET-S/CLA-S (treated with PAC or PAM)	302	23 (8%)	279 (92%)		Ref		Ref	
MET-S/CLA-R (treated with PAM)	109	15 (14%)	94 (86%)		1.94 (0.97–3.86)	0.061	1.90 (0.94–3.86)	0.074
MET-R/CLA-S (treated with PAC)	81	13 (16%)	68 (84%)		**2.32 (1.12–4.81)**	**0.024**	**2.69 (1.25–5.78)**	**0.011**
MET-R/CLA-R	11	0	11 (100%)		N.A		N.A	
Antibiotic resistance				**0.021**				
Fully susceptibility to MET and CLA	302	23 (8%)	279 (92%)		Ref		Ref	
Single resistance to MET or CLA	190	28 (15%)	162 (85%)		**2.10 (1.17–3.76)**	**0.013**	**2.20 (1.22–3.98)**	**0.009**
Double resistance to MET and CLA	11	0	11 (100%)		N.A		N.A	
Tailored triple therapy				0.221				
PPI + AMO + MET (PAM)	218	18 (8%)	200 (92%)		Ref		Ref	
PPI + AMO + CLA (PAC)	285	33 (12%)	252 (88%)		1.46 (0.80–2.66)	0.223	1.59 (0.85–2.96)	0.146
PPI dose per day^g^				0.650				
According to guidelines 2017	262	25 (10%)	237 (90%)		Ref		Ref	
Lower than recommended	232	25 (11%)	207 (89%)		1.15 (0.64–2.06)	0.650	1.31 (0.70–2.45)	0.397
Amoxicillin dose per day^g^				0.500				
According to guidelines 2017	468	49 (10%)	419 (90%)		Ref		Ref	
Lower than recommended	26	1 (4%)	25 (96%)		0.34 (0.05–2.58)	0.298	0.32 (0.04–2.46)	0.273
Drug intake per day				0.220				
Three times per day	97	6 (6%)	91 (94%)		Ref		Ref	
Two times per day	370	38 (10%)	332 (90%)		1.74 (0.71–4.23)	0.225	1.59 (0.61–4.13)	0.339
Use of probiotics				0.940				
Yes	88	9 (10%)	79 (90%)		Ref		Ref	
No	381	40 (11%)	341 (89%)		1.03 (0.48–2.20)	0.941	1.19 (0.54–2.66)	0.665
Adverse events during therapy				0.634				
No	428	46 (11%)	382 (89%)		Ref		Ref	
Yes	52	4 (8%)	48 (92%)		0.69 (0.24–2.01)	0.498	0.81 (0.25–2.58)	0.716
Therapy compliance				** < 0.0001**				
≥ 90% drug intakes	447	32 (7%)	415 (93%)		Ref		Ref	
< 90% drug intakes	30	11 (37%)	19 (63%)		**7.51 (3.29–17.14)**	** < 0.0001**	**6.51 (2.79–15.19)**	** < 0.0001**

Abbreviation: *TTT* tailored triple therapy, *ER* eradication rate, *OR* odd ratio, *PPI* proton pump inhibitor, *AMO* amoxicillin, *CLA* clarithromycin, *MET* metronidazole, *PAC* for treatment regimen with proton pump inhibitor, amoxicillin, clarithromycin, *PAM* for treatment regimen with proton pump inhibitor, amoxicillin, metronidazole, *N.A.* not applicable, *ref.* reference category

^a^*P* values obtained by Mann–Whitney *U*-test for continuous variables, while Pearson’s Chi-square test or Fisher’s exact test for categorical variables as appropriate. Bold *p* values indicate significant differences in the proportion of respective factors between the patient group with eradication failure and the patient group with eradication success by a *p* value ≤ 0.05

^b^Crude odd ratio (OR_crude_) with 95% confidence intervals (95% CI) applied from a univariate logistic regression

^c^*P* values obtained from the Wald Chi-Square Test for the significance of the odd ratio (OR)

^d^Adjusted odds ratios (OR_adj_) with 95% confidence intervals (95% CI) obtained from the multivariable logistic regression adjusted with gender, age in years and country of living

^e^Country distribution was given in supplementary file 2

^f^MET-S/CLA-S: Strains susceptible to both metronidazole and clarithromycin. MET-S/CLA-R: Strains susceptible to metronidazole but resistant to clarithromycin. MET-R/CLA-S: Strains resistant to metronidazole but susceptible to clarithromycin. MET-R/CLA-R: Strains resistant to both metronidazole and clarithromycin

^g^Results were evaluated by comparing the prescribed dose with the standard dosing regimen provided in the updated guidelines 2016 [8]

### Factors associated with treatment failure

**Fig. 2:**
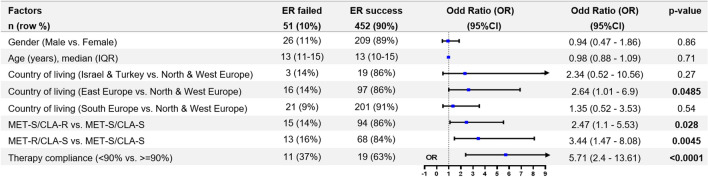
Risk factors for eradication failure applied from multivariable logistic regression among treatment naïve patients (group A) with known antibiotic susceptibility of clarithromycin (CLA) & metronidazole (MET), who received tailored triple therapy (TTT) and completed follow-up, *N* = 503. Abbreviations: *TTT* tailored triple therapy, *ER* eradication rate, *OR* odd ratio, *PPI* proton pump inhibitor, *AMO* amoxicillin, *CLA* clarithromycin, *MET* metronidazole, *MET-S/CLA-S* strains susceptible to both metronidazole and clarithromycin, *MET-S/CLA-R* strains susceptible to metronidazole but resistant to clarithromycin, *MET-R/CLA-S* strains resistant to metronidazole but susceptible to clarithromycin, *MET-R/CLA-R* strains resistant to both metronidazole and clarithromycin. Odds ratios (OR) with 95% confidence intervals (95% CI) obtained from the final multivariable logistic regression are given. *P* values were obtained from the Wald Chi-Square Test for the significance of the odds ratio (OR)

Among treatment naïve patients receiving 2-weeks tailored triple therapy (*n* = 503), we identified in the univariate analysis only antibiotic susceptibility and therapy compliance associated with treatment failure. Other factors, including gender, age, drug dose, number of drug intakes per day, use of probiotics during therapy or reported adverse events, were not significantly associated with treatment failure (Table 3). The multivariable logistic regression showed that eradication failure of tailored triple therapy is three times more likely if the infecting strains are resistant to metronidazole or clarithromycin compared to fully susceptible strains (Fig. 2). Children taking < 90% of prescribed drugs over 14 days had a six times higher risk (OR = 5.89, 95% CI 2.49–13.95, Fig. 3) of treatment failure than children with excellent compliance.

In patients with previously failed therapy (group B), a standard dose compared to the recommended high dose of amoxicillin was associated with five times increased risk for treatment failure (OR = 5.04, 95% CI 1.09–23.28) (Supplementary Files 2 & 8). Low versus high compliance increased the estimated risk for unsuccessful therapy (OR = 12.93, 95% CI 1.93–86.75, Supplementary File 8).

### Adverse events during therapy

In the final analysis set, adverse events during therapy were reported in 12% (96/822), mainly abdominal pain (*n* = 54), diarrhoea (*n* = 28), nausea (*n* = 26) and vomiting (*n* = 22). Of those, 19% (18/96) failed therapy (*A*: 12%, *n* = 81; *B*: 53%, *n* = 15).

### Sensitivity analysis

In seven (1.4%) of 503 patients, the caring physician chose the wrong antibiotics: twice PAC instead of PAM in single clarithromycin-resistant strains, three times PAM instead of PAC in single metronidazole-resistant strains and twice PAC with high-dose amoxicillin instead of PAM in infections with double-resistant strains. In patients infected with single-resistant strains, the sensitivity analysis revealed marginal differences compared to the real-life situation with an eradication rate of 86% (*n* = 185) versus 85% (*n* = 190), respectively (Supplementary File 9A and Fig. 1A). The multivariable logistic regression showed that the risk of eradication failure of tailored triple therapy for 2 weeks was still 2.74 times more likely in children infected with *H. pylori* strains resistant to metronidazole or clarithromycin compared to fully susceptible strains, *p* = 0.0037.

## Discussion

Our findings confirm that a primary eradication rate of at least 90% is feasible in clinical practice with a 2-week triple therapy tailored to antibiotic susceptibility results giving higher doses as recommended in the recent ESPGHAN/NASPGHAN guidelines [8]. Being infected with an *H. pylori* strain resistant to clarithromycin or metronidazole and an intake of < 90% of prescribed drugs are significant risk factors for primary treatment failure of tailored triple therapy.

### Antibiotic resistance of *H. pylori* strains

Our registry data provide a long-term and comprehensive surveillance of antibiotic resistance of *H. pylori* strains in children living in Europe. Our first survey covered 1999–2002 [18], the second 2013–2016 [17], and the current 2017–2020, reporting all consecutive *H. pylori*-infected patients from the paediatric centres. For treatment naïve children, selection bias regarding antibiotic susceptibility is unlikely. Comparing the primary resistance to clarithromycin, we noticed a slight increase over time (20%, 24.8%, and 25%, respectively), while resistance to metronidazole decreased (23%, 20.9% and 17.7%, respectively). Several recent European intervention programmes restricting macrolide consumption, particularly for respiratory tract infections in children [9, 19, 20], may have prevented a further increase in the clarithromycin resistance rate from the second to the third survey. While macrolides are often prescribed to children and may induce resistance to *H. pylori* strains, metronidazole is rarely used in paediatrics. Therefore, children most likely acquire a metronidazole-resistant strain, with their mothers being the main source of infection. In all three periods, resistance to clarithromycin and/or metronidazole was significantly more frequent in strains from children after failed therapy compared to treatment naïve patients [17, 18].

### Sampling error and treatment failure

The wide availability of antibiotic susceptibility testing in Europe allows us to evaluate the eradication success of 2-weeks tailored triple therapy for four antibiotic susceptibility sub-groups: Fully susceptible, single resistant to clarithromycin, single resistant to metronidazole and double resistant. Of children compliant with drugs, 5% failed the first attempt if infected with fully susceptible strains (Fig. 1B), while the failure rate was 12% in children infected with single-resistant strains (Fig. 1B). Children infected with single-resistant strains (*n* = 190) had a two to three times higher risk for treatment failure despite treatment with antibiotics; they were susceptible to compared to those with fully susceptible strains (Fig. 2). This significant difference remained after excluding five cases where the physicians had chosen the wrong antibiotics (Supplementary File 9A). We hypothesize that the main cause of treatment failure despite therapy tailored to antibiotic susceptibility is missed mixed infections. In a previous study on 83 infected children, we evaluated gastric biopsies taken at the same endoscopy, one biopsy each by E-testing in two different laboratories and the third one by in situ hybridisation [21]. In 11 patients (13%), we found discrepant results regarding clarithromycin resistance between the applied methods indicating mixed infections with the co-existence of a clarithromycin-susceptible and a clarithromycin-resistant strain which was obviously not evenly distributed in the stomach [21, 22]. Considering the result of antibiotic susceptibility based on only one biopsy leads to an underestimation of around 5% of clarithromycin resistance in the sub-groups “fully susceptible” and “single metronidazole resistance”. Missing a clarithromycin-resistant strain has a higher clinical impact because PAC has a low eradication rate in clarithromycin-resistant infections, while in vitro metronidazole resistance may be overcome in vivo by a higher drug dose and longer duration of therapy [11, 13, 23]. By comparing PAC to PAM, our hypothesis supports the findings of a 4–5% lower eradication rate in children with single resistance and children with fully susceptible strains (Table 2, Supplementary File 6). Moreover, 95% eradication is obtained only in children with fully susceptible strains taking > 90% of prescribed drugs.

We conclude from our findings to take at least two biopsies in the antrum and corpus for culture if *H. pylori* infection is macroscopically suspected (e.g. antral nodularity, peptic lesions) or after failed therapy to improve the success rate for culture [24] and to decrease the risk of missing resistant strains. The recent guidelines recommend obtaining at least six gastric biopsies, thereof four for histopathology and the remaining two for culture and rapid urase test [8]. In our cohort, six biopsies were documented in only 25% of patients, giving room for improvement. Moreover, we suggest preferring PAM over PAC to treat the fully susceptible group in case of missed clarithromycin-resistant strains by sampling error.

### Optimized drug dosing regimens

Sufficient acid suppression is crucial for effectiveness because, at high pH, the bacteria enter their replicative state and become susceptible to amoxicillin and clarithromycin [23]. Children around puberty have higher CYP2C19 enzyme activity to metabolize PPIs, including (es)-omeprazole; hence, they may need higher doses per kg body weight than adults for equivalent acid suppression [25, 26]. The updated paediatric guidelines recommend higher PPI doses for all regimens (Supplementary File 2) [8]. For different reasons, this recommendation was followed in only half of the patients (e.g. national regulations or high costs). The acid-suppressive capacity of PPIs may be negatively affected by under-dosing, by taking the drug not prior but with or after meals, by larger dosing intervals (daily dose divided into two versus three intakes), and by genetic polymorphism of the hepatic CYP2C19 enzyme activity [25]. The latter determines fast (70% of Caucasians), intermediate 25–30% of Caucasians) and slow metabolizer (2–5% of Caucasians) [27]. Esomeprazole is less susceptible to degradation by fast metabolizers than pantoprazole, resulting in a higher and better predictable acid-inhibitory effect. Therefore, esomeprazole-based tailored triple therapy was prescribed to 60% of our treatment-naïve patients, followed by omeprazole (32%), while lansoprazole and pantoprazole were used by only 2% and 6%, respectively. Randomized controlled trials may be needed to clarify the role of PPI type and dosing on the success of 2-weeks tailored triple therapy.

In contrast to PPI dosing, we found a four times higher risk of treatment failure using standard compared to a high amoxicillin dose. We could previously show that in children infected with a double-resistant strain, PAM with high-dose amoxicillin was successful in 75% (22/45) of children compliant with the 2-week therapy [28] (Supplementary File 7). The benefit of high-dose amoxicillin in the tailored triple therapy was reported in reducing the emergence of resistance to co-antibiotics [6]. Thus, using a higher amoxicillin dose may further increase the effectiveness of clarithromycin or metronidazole in tailored triple therapy.

### Therapy compliance

Poor therapy compliance is a significant risk factor for treatment failure. Kotilea et al. 2017 [16] demonstrated that with high compliance, defined as more than 90% intake of prescribed doses, a success rate of 89.9% was achieved, while patients with lower adherence reached only 36.8%. To improve compliance, the *H. pylori* working group developed an information leaflet for parents and children on the importance of strict drug intake to successfully treat the infection (links: https://www.espghan.org/knowledge-center/education/H-Pylori-Patient-Parent-Guide) [29].

### Other therapy regimens

Based on previous publications of our group, a 10-days sequential therapy was given as an option in the current guidelines, but only to treat patients infected with fully susceptible strains [3, 8, 14]. In the present cohort, sequential therapy failed the treatment goal of 90% even in these patients (Table 2). Furthermore, like in concomitant regimens, sequential therapy contains three antibiotics (amoxicillin, clarithromycin and metronidazole), in which one of them does not contribute to eradication success. According to antibiotic stewardship principles, therapies using antibiotic combinations assuming the infection will be susceptible to at least one [5, 6] should be abandoned. Since we achieve eradication of 90% and higher with 2-weeks triple therapy tailored to antibiotic susceptibility, we suggest that neither sequential therapy nor concomitant regimens should be prescribed to treat *H. pylori* infection in children.

Bismuth-based regimens achieved a high cure rate as second-line therapy, including treatment of patients infected with double-resistant strains. For older adolescents, PPI with a capsule containing bismuth-subcitrate, tetracycline and metronidazole for compassionate use would be an alternative [30].

### Strengths and limitations

Our registry attained unique and comprehensive surveillance over four years on a large number of consecutive *H. pylori*-infected paediatric patients in Europe with complete data on demographics, clinical and endoscopic presentation, antibiotic resistance, treatment, and follow-up. The unbalanced number of children included from participating centres and countries reflects the different prevalence of paediatric *H. pylori* infection in European countries and the patient population care in the different centres. This impacts the reported primary antibiotic resistance in treatment naïve patients since we previously showed that country of living and the mother’s country of birth has a major impact on the antibiotic resistance rates towards clarithromycin and metronidazole [17]. However, the uneven recruitment should not introduce a bias towards our reported results of treatment success rates because children were treated with 2-weeks antibiotics tailored to antibiotic susceptibility results and with dosing recommended by guidelines [8]. The recommendations are not difficult to follow since in only 7 (1.4%) children, physicians made a mistake by prescribing an antibiotic the child was resistant to (not guideline conform). All children underwent endoscopy because of symptoms or underlying disease (e.g. eosinophilic esophagitis, inflammatory bowel disease, or celiac disease). Therefore, we have an enrichment (7.1%) of these co-morbidities in our cohort compared to the general population, but this should also not introduce a bias with respect to eradication success of tailored triple therapy for 2 weeks. Unlike the European registry on *H. pylori*-infected adults [31], we provided feedback annually to participating centres, including suggestions to improve adherence to guidelines and treatment success.

Our study has several limitations. First, the antibiotic susceptibility testing was performed locally and not in a central laboratory due to the complexity of sample transport and financial restriction. E-test was the most common tool used for susceptibility testing, and antibiotic resistance breakpoints were unified in the laboratories by applying the guidelines of the European Committee of Antibiotic Susceptibility Testing (EUCAST). Second, we cannot exclude recall bias for any previous *H. pylori* eradication treatment, especially in patients with migration backgrounds and language barriers. Third, concerning compliance, we relied on parents’ reporting to their physicians: medications completely taken (100% compliance), one day (90%), 2–3 days (70–90%), ≥ 4 days left out (≤ 70%). The estimated therapy compliance in our cohort was high since adherent patients are more likely to return for follow-up visits to monitor the success of therapy than patients not adhering to therapy. Fourth, loss to follow-up occurred in one-third of all treated patients. In some countries or clinical settings, monitoring visits at the outpatient clinic are either impossible or not reimbursed by health insurance; other reasons include long travel distances to the hospital or missed appointments. However, this should not influence the results of per-protocol analysis.

### Practical implications for clinical routine

In the absence of bismuth-based combination drugs, triple therapy tailored to antibiotic susceptibility for 2 weeks with drug doses as recommended in the ESPGHAN/NASPGHAN guidelines is currently the best option to treat *H. pylori*-infected children and adolescents with a primary success rate of ≥ 90% following the principles of the antibiotic stewardship program.

Our data suggest that taking two or more biopsies (antrum and corpus) for antibiotic susceptibility testing may increase the chance to detect clarithromycin-resistant bacterial strains in case of mixed infection, having an uneven distribution of clarithromycin-suseptible and clarithromycin-resistant *H. pylori* strains in the stomach. Tailored triple therapy combining PPI, amoxicillin with metronidazole (PAM) should be preferred over the combination with clarithromycin (PAC) to treat patients with fully susceptible strains in regions or populations known for high clarithromycin resistance. Applying recommended PPI and antibiotic dosing regimens [8] (Supplementary File 2) optimizes the effectiveness of tailored triple therapy. Patient education is crucial for high adherence to therapy. These measures improve treatment success and reduce later complications and costs.

## Conclusion

In conclusion, 2-weeks triple therapy with PPI, amoxicillin and clarithromycin or metronidazole tailored to antibiotic susceptibility with optimized doses remains highly effective as the first-line therapy in *H. pylori*-infected children and adolescents. An anticipated primary eradication rate of at least 90% will reduce the need for repeated or unnecessary antibiotic exposures, the risk for long-term adverse effects on the child’s microbiota, and the development of resistant strains. Whether obtaining two or more gastric biopsies for antibiotic susceptibility testing may further increase eradication rate of tailored triple therapy needs to be investigated in future studies. Guideline-conform management following the antibiotic stewardship program principles will contribute to reducing global antibiotic resistance.

Practical guidance should be provided to paediatric gastroenterologists, paediatricians, and general practitioners, encouraging them to follow guidelines, including consequent noninvasive monitoring for treatment success. Systematic surveillance of antibiotic resistance and continuous centre monitoring is fundamental to improve the quality of care in *H. pylori*-infected patients.

## Supplementary Information

Below is the link to the electronic supplementary material.

Supplementary file1 (DOCX 287 KB)
