# Supplementary material for: Management of Helicobacter pylori infection in paediatric patients in Europe: results from the EuroPedHp Registry
Source: Infection. 2022 Nov 3;51(4):921–34. doi: 10.1007/s15010-022-01948-y (PMC10352155; doi:10.1007/s15010-022-01948-y)
Supplement: Supplementary file 1 — Supplementary file1 (DOCX 287 KB) [file 15010_2022_1948_MOESM1_ESM.docx]

**Supplementary file legends**

[**Supplementary file 1:** Countries assigned to four geographical regions 2](#_Toc116596894)

[**Supplementary file 2:** Dosing regimens recommended in the updated ESPGHAN/NASPGHAN guidelines; e-publication in 2016 [1] 3](#_Toc116596895)

[**Supplementary file 3:** Detailed explanation of statistical analysis 4](#_Toc116596896)

[**Supplementary file 4:** Flowchart of the study population 5](#_Toc116596897)

[**Supplementary file 5:** Number of included patients per country from 2017 to 2020: of 1543 included patients in the EuroPedHp registry, 1263 were treated, thereof 873 completed follow-up 6](#_Toc116596898)

[**Supplementary file 6:** Eradication rate (ER) of PAC vs PAM in patients with fully susceptible strains & single resistant strains: A) final analysis set (FAS) and B) Per protocol (PP) population 7](#_Toc116596899)

[**Supplementary file 7:** Risk factors for eradication failure of tailored triple therapy (TTT) among patients after failed therapy (group B), N=69 8](#_Toc116596900)

[**Supplementary file 8:** Risk factors for eradication failure applied from multivariable logistic regression in patients after failed therapy (group B) with known antibiotic susceptibility to clarithromycin (CLA) & metronidazole (MET); all received tailored triple therapy (TTT) and completed follow-up, N=69 9](#_Toc116596901)

[**Supplementary file 9 A and B:** Eradication rate (ER) of tailored triple therapy (TTT) in patients with fully susceptible vs single resistant strains. A) in full analysis set (FAS) B) Per protocol (PP) population obtained from the sensitivity analysis 10](#_Toc116596902)

**Supplementary file 1:** Countries assigned to four geographical regions

| **Country regions** | Countries |
| --- | --- |
| **Northern/Western Europe** |  |
| Northern Europe | Sweden, Norway, Finland |
| Western Europe | UK, England, Ireland, Scotland, France, Netherlands, Belgium, Germany, Austria, Switzerland, Luxembourg |
| **Southern Europe** | Portugal, Spain, Italy, Greece |
| **Eastern Europe** | Albania, Bosnia, Kosovo, Serbia, Macedonia, Romania, Slovenia, Moldova, Ukraine, Russia, Chechnya, Lithuania, Poland, Hungary, Czech Republic, Slovakia, Croatia, Azerbaijan, Moldavia, Rumania |
| **Asia, Africa, America, and the Middle East** | Angola, Guinea, Somalia, Senegal, Ghana, Cabo to Verde, Guinea, Guinea to Bissau, Madagascar, South Africa, Libya, Morocco, Tunisia, Algeria, Ethiopia, Eritrea, Republic Dominican, China, Mongolia, Thailand, Vietnam, New Guinea, Iran, Iraq, Iraq, Syria, Egypt, Turkey, Israel, Armenia, Bangladesh, Afghanistan, Afghanistan, Kazakhstan, Kazakhstan, Azerbaijan, India, Nepal, Azerbaijan, Canada, USA, US, Colombia, Chile, Peru, Ecuador, Bolivia Argentina, Brazil, Uruguay, Paraguay, Argentina, Australia. |

**Supplementary file 2:** Dosing regimens recommended in the updated ESPGHAN/NASPGHAN guidelines; e-publication in 2016 [1]

| 1. **Standard dosing regimen** | | | | |
| --- | --- | --- | --- | --- |
| **Drug** | **Bodyweight range** | **Morning dose, mg** | **Evening dose, mg** | **Daily total dose, mg** |
| **PPI Es(omeprazole)** | 15 - 24 kg | 20 mg | 20 mg | 40 mg |
|  | 25 - 34 kg | 30 mg | 30 mg | 60 mg |
|  | > 35 kg | 40 mg | 40 mg | 80 mg |
| **Amoxicillin**  **(AMO)** | 15 - 24 kg | 500 mg | 500 mg | 1000 mg |
|  | 25 - 34 kg | 750 mg | 750 mg | 1500 mg |
|  | > 35 kg | 1000 mg | 1000 mg | 2000 mg |
| **Clarithromycin (CLA)** | 15 - 24 kg | 250 mg | 250 mg | 500 mg |
|  | 25 - 34 kg | 500 mg | 250 mg | 750 mg |
|  | > 35 kg | 500 mg | 500 mg | 1000 mg |
| **Metronidazole (MET)** | 15 - 24 kg | 250 mg | 250 mg | 500 mg |
|  | 25 - 34 kg | 500 mg | 250 mg | 750 mg |
|  | > 35 kg | 500 mg | 500 mg | 1000 mg |
| 1. **High dosing regimen for amoxicillin** | | | | |
| **Drug** | **Bodyweight range** | **Morning dose, mg** | **Evening dose, mg** | **Daily total dose, mg** |
| **Amoxicillin (AMO)** | 15 - 24 kg | 750 mg | 750 mg | 1500 mg |
|  | 25 - 34 kg | 1000 mg | 1000 mg | 2000 mg |
|  | > 35 kg | 1500 mg | 1500 mg | 3000 mg |

Abbreviation: PPI proton pump inhibitor

Adapted with permission from Jones NL, Koletzko S et al.; ESPGHAN, NASPGHAN. Joint ESPGHAN/NASPGHAN Guidelines for the Management of Helicobacter pylori in Children and Adolescents (Update 2016). J Pediatr Gastroenterol Nutr. 2017 Jun;64(6):991-1003.

**Supplementary file 3:** Detailed explanation of statistical analysis

Results were presented in median and interquartile range (IQR) from 25% quartile to 75% quartile for continuous variables and in frequency (n) and percentage (%) for categorical variables. To determine statistically significant differences between groups, we performed Mann-Whitney-U-test for continuous variables, while Pearson's Chi-square test or Fisher's exact test for categorical variables where appropriate. All statistical tests were assessed with two-sided significance levels of 5%.

A univariate logistic analysis was performed to determine potential risk factors for treatment failure. Crude odd ratios (OR_crude_) with 95% confidence intervals (95% CI) applied from a univariate logistic regression were shown. Adjusted odds ratios (OR_adj_) with 95% confidence intervals (95% CI) were obtained from the multivariable logistic regression adjusted with gender, age in years and country of living.

All variables associated with treatment failure at p-value ≤0.25 in the univariate analysis were considered in the multivariable logistic regression. Using the same samples as in the univariate analysis, the final multivariable logistic models were selected using backward elimination and adjusted for gender and age (in years). Logistic analyses were performed with complete datasets with no missing values in covariates. Interaction and effect modification between considered variables and gender or age were examined parallel to backward elimination. Estimated odds ratio (OR) and 95% CI were reported. P-values from the Wald Chi-Square Test determine the significance of the odd ratio (OR). Statistical significance was considered at p ≤ 0.05.


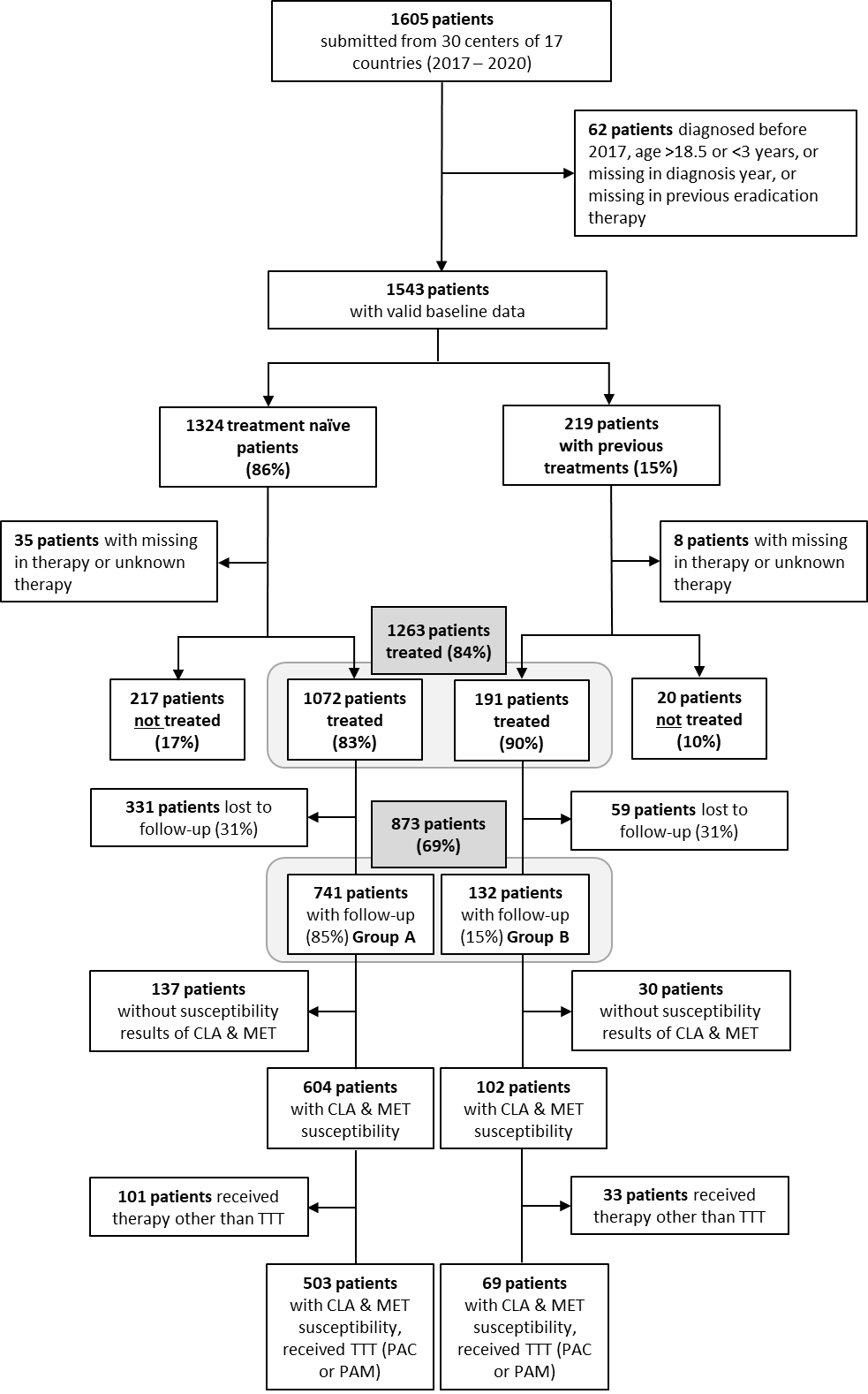


**Supplementary file 4:** Flowchart of the study population

Abbreviation: TTT tailored triple therapy; CLA clarithromycin; MET metronidazole; PAC for treatment regimen with proton pump inhibitor, amoxicillin, clarithromycin; PAM for treatment regimen with proton pump inhibitor, amoxicillin, metronidazole; Group A (treatment naïve patients) and group B (patients after failed therapy).

**Supplementary file 5:** Number of included patients per country from 2017 to 2020: of 1543 included patients in the EuroPedHp registry, 1263 were treated, thereof 873 completed follow-up

**Supplementary file 6:** Eradication rate (ER) of PAC vs PAM in patients with fully susceptible strains & single resistant strains: A) final analysis set (FAS) and B) Per protocol (PP) population

Abbreviation: ER eradication rate; CLA clarithromycin; MET metronidazole; PAC for treatment regimen with proton pump inhibitor, amoxicillin, clarithromycin; PAM for treatment regimen with proton pump inhibitor, amoxicillin, metronidazole.

ER% represents eradication rate (ER) in per cent (%) as the proportion of all patients treated successfully with a confirmed negative test after completed treatment relative to all patients treated in a specific sub-group.

P-values were obtained from Pearson’s Chi-square test to determine the significant difference in eradication rate (ER) between the patient group that received PAC vs PAM.

**Supplementary file 7:** Risk factors for eradication failure of tailored triple therapy (TTT) among patients after failed therapy (group B), N=69

| **Factors, n (row per cent %)** | **N** | **ER failed**  **n (%)** | **ER success**  **n (%)** | **p-value ^a)^** | **OR_crude_ ^b)^ (95%CI)** | **p-value ^c)^** | **OR_adj_ ^d)^ (95%CI)** | **p-value ^c)^** |
| --- | --- | --- | --- | --- | --- | --- | --- | --- |
| **Gender** |  |  |  |  |  |  |  |  |
| Female | 44 | 15 (34%) | 29 (66%) | 0.145 | ref. |  |  |  |
| Male | 25 | 13 (52%) | 12 (48%) |  | 2.09 (0.77 - 5.71) | 0.148 |  |  |
| **Age (years), median (IQR)** | 69 | 12 (9 - 14) | 13 (10 - 15) | 0.065 | 0.91 (0.79 - 1.05) | 0.197 |  |  |
| **Country of living ^e)^** |  |  |  | 0.163 |  |  |  |  |
| Northern/Western Europe | 15 | 5 (33%) | 10 (67%) |  | ref. |  |  |  |
| Southern Europe | 26 | 9 (35%) | 17 (65%) |  | 1.06 (0.28 - 4.06) | 0.934 |  |  |
| Eastern Europe | 13 | 4 (31%) | 9 (69%) |  | 0.89 (0.18 - 4.38) | 0.885 |  |  |
| Israel & Turkey | 15 | 10 (67%) | 5 (33%) |  | 4.00 (0.88 - 18.26) | 0.074 |  |  |
| **Susceptibility sub-groups ^f)^** |  |  |  | 0.066 |  |  |  |  |
| MET-S / CLA-S  (treated with PAC or PAM) | 34 | 12 (35%) | 22 (65%) |  | ref. |  | ref. |  |
| MET-S / CLA-R  (treated with PAM) | 21 | 6 (29%) | 15 (71%) |  | 0.73 (0.23 - 2.39) | 0.606 | 1.23 (0.26 - 5.83) | 0.791 |
| MET-R / CLA-S  (treated with PAC) | 10 | 7 (70%) | 3 (30%) |  | 4.28 (0.93 - 19.65) | 0.062 | **6.36 (1.003 - 40.38)** | **0.0496** |
| MET-R / CLA-R | 4 | 3 (75%) | 1 (25%) |  | 5.50 (0.51 - 58.83) | 0.159 | **18.07 (1.19 - 274.8)** | **0.037** |
| **Antibiotic resistance** |  |  |  | 0.355 |  |  |  |  |
| Fully susceptibility | 34 | 12 (35%) | 22 (65%) |  | ref. |  | ref. |  |
| Single resistance | 31 | 13 (42%) | 18 (58%) |  | 1.32(0.49 - 3.61) | 0.583 | 2.40 (0.66 - 8.81) | 0.187 |
| Double resistance | 4 | 3 (75%) | 1 (25%) |  | 5.50 (0.51 - 58.77) | 0.159 | **15.82 (1.09 - 229.9)** | **0.043** |
| **Tailored triple therapy** |  |  |  | 0.305 |  |  |  |  |
| PPI + AMO + AMO (PAM) | 42 | 15 (36%) | 27 (64%) |  | ref. |  | ref. |  |
| PPI + AMO + CLA (PAC) | 27 | 13 (48%) | 14 (52%) |  | 1.67 (0.63 - 4.47) | 0.306 | 1.03 (0.32 - 3.35) | 0.955 |
| **PPI dose per day ^g)^** |  |  |  | **0.004** |  |  |  |  |
| According to guidelines 2016 | 40 | 22 (55%) | 18 (45%) |  | ref. |  | ref. |  |
| Lower than recommended | 29 | 6 (21%) | 23 (79%) |  | **0.21 (0.07 - 0.64)** | **0.006** | **0.20 (0.05 - 0.72)** | **0.014** |
| **Amoxicillin dose per day ^g)^** |  |  |  | 0.073 |  |  |  |  |
| High dose acc. guidelines 2016 | 24 | 6 (25%) | 18 (75%) |  | ref. |  | ref. |  |
| Lower than recommended | 45 | 22 (49%) | 23 (51%) |  | 2.87 (0.96 - 8.56) | 0.059 | **3.94 (1.01 - 15.23)** | **0.048** |
| **Drug intake per day** |  |  |  | 0.074 |  |  |  |  |
| Three times per day | 6 | 0 | 6 (100%) |  | N.A. |  | N.A. |  |
| Two times per day | 61 | 26 (43%) | 35 (57%) |  | N.A. |  | N.A. |  |
| **Use of probiotics** |  |  |  | 0.691 |  |  |  |  |
| Yes | 7 | 2 (29%) | 5 (71%) |  | ref. |  | ref. |  |
| No | 59 | 25 (42%) | 34 (58%) |  | 1.84 (0.33 - 10.25) | 0.488 | 0.94 (0.13 - 6.77) | 0.954 |
| **Adverse events during therapy** |  |  |  | 0.436 |  |  |  |  |
| No | 53 | 21 (40%) | 32 (60%) |  | ref. |  | ref. |  |
| Yes | 7 | 4 (57%) | 3 (43%) |  | 2.03 (0.41 - 10.01) | 0.384 | 2.56 (0.40 - 16.42) | 0.323 |
| **Therapy compliance** |  |  |  | 0.021 |  |  |  |  |
| ≥ 90% drug intakes | 54 | 17 (31%) | 37 (69%) |  | ref. |  | ref. |  |
| < 90% drug intakes | 9 | 7 (78%) | 2 (22%) |  | **7.62 (1.43 - 40.59)** | **0.017** | **10.58 (1.41 - 79.28)** | **0.022** |

Analyses were performed with complete datasets with no missing values in covariates.

Abbreviation: TTT tailored triple therapy; ER eradication rate; OR odd ratio; PPI proton pump inhibitor; AMO amoxicillin; CLA clarithromycin; MET metronidazole; PAC for treatment regimen with proton pump inhibitor, amoxicillin, clarithromycin; PAM for treatment regimen with proton pump inhibitor, amoxicillin, metronidazole; N.A. not applicable; ref. reference category.

^a)^ P-values obtained by Mann–Whitney-U-test for continuous variables, while Pearson’s Chi-square test or Fisher’s exact test for categorical variables as appropriate. Bold p-values indicate significant differences in the proportion of respective factors between the patient group with eradication failure and the patient group with eradication success by a p-value ≤0.05.

^b)^ Crude odd ratio (OR_crude_) with 95% confidence intervals (95% CI) applied from a univariate logistic regression.

^c)^ P-values obtained from the Wald Chi-Square Test for the significance of the odd ratio (OR).

^d)^ Adjusted odds ratios (OR_adj_) with 95% confidence intervals (95% CI) obtained from the multivariable logistic regression adjusted with gender, age in years and country of living.

^e)^ Country distribution was given in supplementary file 2.

^f)^ MET-S/CLA-S: Strains susceptible to both metronidazole and clarithromycin. MET-S/CLA-R: Strains susceptible to metronidazole but resistant to clarithromycin. MET-R/CLA-S: Strains resistant to metronidazole but susceptible to clarithromycin. MET-R/CLA-R: Strains resistant to both metronidazole and clarithromycin. ^g)^ Results were evaluated by comparing the prescribed dose with the standard dosing regimen provided in the updated guidelines 2016 [1].


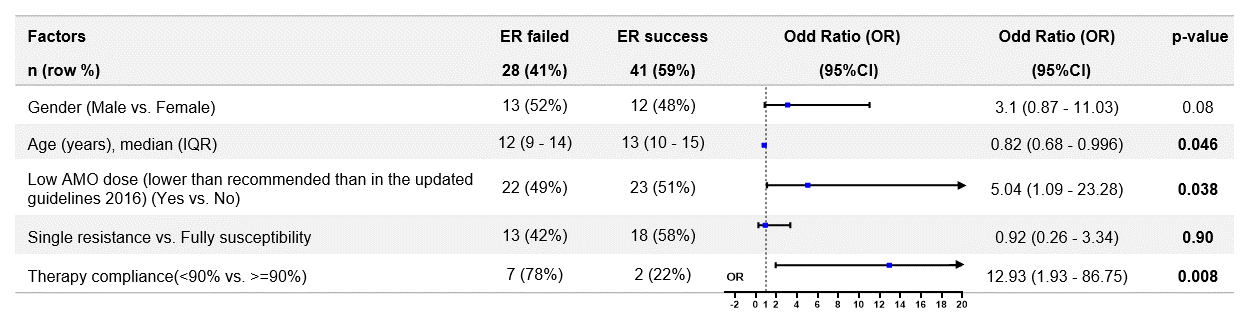
**Supplementary file 8:** Risk factors for eradication failure applied from multivariable logistic regression in patients after failed therapy (group B) with known antibiotic susceptibility to clarithromycin (CLA) & metronidazole (MET); all received tailored triple therapy (TTT) and completed follow-up, N=69

Abbreviation: TTT tailored triple therapy; ER eradication rate; OR odd ratio; PPI proton pump inhibitor; AMO amoxicillin; CLA clarithromycin; MET metronidazole.

Fully susceptibility represents strains susceptible to both clarithromycin and metronidazole, while single resistance determines strains resistant to clarithromycin or metronidazole.

Odds ratios (OR) with 95% confidence intervals (95% CI) obtained from the final multivariable logistic regression are given. P-values were obtained from the Wald Chi-Square Test for the significance of the odds ratio (OR).

**Supplementary file 9 A and B:** Eradication rate (ER) of tailored triple therapy (TTT) in patients with fully susceptible vs single resistant strains. A) in full analysis set (FAS) B) Per protocol (PP) population obtained from the sensitivity analysis

Abbreviation: ER eradication rate; CLA clarithromycin; MET metronidazole; PAC for treatment regimen with proton pump inhibitor, amoxicillin, clarithromycin; PAM for treatment regimen with proton pump inhibitor, amoxicillin, metronidazole.

ER% represents eradication rate (ER) in per cent (%) as the proportion of all patients treated successfully with a confirmed negative test after completed treatment relative to all patients treated in a specific sub-group.

P-values were obtained from Pearson’s Chi-square test to determine the significant difference in eradication rate (ER) between the patient group that received PAC vs PAM.

**Reference**

1. Jones NL, Koletzko S, Goodman K, Bontems P, Cadranel S, Casswall T, et al. Joint ESPGHAN/NASPGHAN Guidelines for the Management of Helicobacter pylori in Children and Adolescents (Update 2016). J Pediatr Gastroenterol Nutr. 2017;64(6):991-1003. <https://doi.org/10.1097/MPG.0000000000001594>.
